# Risk factors for contacts between wild boar and outdoor pigs in Switzerland and investigations on potential *Brucella suis* spill-over

**DOI:** 10.1186/1746-6148-8-116

**Published:** 2012-07-20

**Authors:** Natacha Wu, Carlos Abril, Andreas Thomann, Eleonore Grosclaude, Marcus G Doherr, Patrick Boujon, Marie-Pierre Ryser-Degiorgis

**Affiliations:** 1 Centre for Fish and Wildlife Health (FIWI), Institute of Animal Pathology, Vetsuisse Faculty, University of Bern, Bern, Switzerland; 2 Institute of Veterinary Bacteriology, Centre for Zoonoses, Bacterial Animal Diseases and Antimicrobial Resistance (ZOBA), Vetsuisse Faculty, University of Bern, Bern, Switzerland; 3 Veterinary Public Health Institute, Vetsuisse Faculty, University of Bern, Bern, Switzerland; 4 Service de la Consommation et des Affaires Vétérinaires, Geneva, Switzerland; 5 Present address: Institut Galli-Valerio, Laboratoire d’analyses vétérinaires, Lausanne, Switzerland; 6 Suisselab AG, Schützenmattstrasse 10, 3052, Zollikofen, Switzerland

**Keywords:** *Brucella suis*, Brucellosis, Interactions, Outdoor pigs, Risk factors, Spill-over, Wild boar

## Abstract

**Background:**

Due to the parallel increase of the number of free-ranging wild boar and domestic pigs reared outdoor, the risk that they interact has become higher. Contacts with wild boar can be the origin of disease outbreaks in pigs, as it has been documented for brucellosis in some European countries. This study aimed at quantifying the occurrence of contacts between wild boar and outdoor domestic pigs in Switzerland, and identifying risk factors for these contacts. Furthermore, exposed pigs were tested for pathogen spill-over, taking *Brucella suis* as an example because *B. suis* is widespread in Swiss wild boar while domestic pigs are officially free of brucellosis.

**Results:**

Thirty-one percent of the game-wardens and 25% of the pig owners participating to a country-wide questionnaire survey reported contacts, including approaches of wild boar outside the fence, intrusions, and mating. Seventeen piggeries (5%) reported the birth of cross-bred animals. Risk factors for contacts identified by a uni- and multivariable logistic regression approach were: distance between pigs enclosure and houses, proximity of a forest, electric fences, and fences ≤ 60 cm. Pigs of the Mangalitza breed were most at risk for mating with wild boar (births of cross-bred animals). Blood and tissues of 218 outdoor pigs from 13 piggeries were tested for an infection with *Brucella suis*, using rose bengal test, complement fixation test, and an IS*711*-based real-time PCR. One piggery with previous wild boar contacts was found infected with *B. suis*, however, epidemiological investigations failed to identify the direct source of infection.

**Conclusions:**

Results show that interactions between wild boar and outdoor pigs are not uncommon, pointing at the existing risk of pathogen spill-over. Provided data on risk factors for these interactions could help the risk-based implementation of protection measures for piggeries. The documentation of a brucellosis outbreak in pigs despite the freedom-of-disease status underlines the importance of improving pathogen surveillance strategies and increasing disease awareness of farmers and veterinary practitioners.

## Background

The wild boar (*Sus scrofa*) population is expanding in Europe [1-3]. Rearing domestic pigs outdoor also shows an increasing trend [4]. Although this type of animal husbandry provides a better quality of life for pigs [5,6], it exposes them to a higher risk of contact with wild boar, which can carry pathogens with various routes of transmission [7].

Important air-born pig diseases include porcine reproductive and respiratory syndrome (PRRS) and enzootic pneumonia (EP; [8]). In contrast to the PRRS virus, *Mycoplasma hyopneumoniae* (causing EP) seems to be widespread in Swiss wild boar, which are considered a potential reservoir for this pathogen [9]. Classical swine fever (CSF) virus and *Mycobacterium bovis* (causing tuberculosis) are transmitted by indirect or direct close contact [2,10]. These pathogens are currently not an issue in Switzerland [11,12] but they could be introduced by wild boar emigrating from neighbouring countries [13-15]. *Sarcoptes scabiei* (causing sarcoptic mange) is transmitted by close contact [16]. While efforts are done to eradicate the mite from Swiss piggeries [17], infestations have been recently diagnosed in wild boar (FIWI, unpublished observations). In wild boar, Aujeszky's disease (AD) virus and *Brucella suis* (causing brucellosis) are transmitted mainly by the venereal route [18,19]. While AD virus infection is rare in Swiss wild boar [11], nearly 30% of them are carrier of *B. suis*, including adults with genital organ infection [1].

The objectives of this study were (a) to document the occurrence of contacts between wild boar and outdoor pigs in Switzerland, (b) to identify risk factors for such contacts in order to propose risk-adapted protection measures for piggeries, and (c) to test exposed pigs for a possible pathogen spill-over, taking *B. suis* as an example. This pathogen was selected because infections have been well documented in Swiss wild boar [1,11,20], while domestic pigs are officially free of brucellosis [21]. A re-emergence of brucellosis in outdoor pigs has been observed in France following intrusions of and/or mating with wild boar [22], indicating a substantial risk of losing the “free from disease” status in Switzerland if close contacts between wild boar and pigs occur. Furthermore, since transmission of *B. suis* mostly requires the closest type of contact (mating), *B. suis* spill-over would suggest that transmission of all other pathogens is possible.

Here we report different types of interactions between wild boar and outdoor pigs and show that risk factors are not only related to wild boar presence, piggery location and fence characteristics but also include pig breed. We additionally report field investigations performed in the frame of a brucellosis outbreak in domestic pigs with a history of contacts with wild boar.

## Results

### Contacts between wild boar and pigs

#### Questionnaire surveys among game-wardens and pig farmers

Thirty-one percent (26/82) of the game-wardens who participated to the study observed contacts at least once between 1995 and 2008, and additionally before 1995 for four of them. Overall, contacts of any category (categories 1–4, see definitions in Methods) were reported in 13 Swiss cantons. Observations included tracks but also intrusions (N = 10) and domestic sows with cross-bred piglets (N = 9). Births of cross-bred animals were reported from 7/13 cantons.

Of the 322 pig farmers who replied the questionnaire, 175 had fattening/finishing pigs, 77 had breeding pigs, 59 kept both types of animals, one held pigs as a hobby, and no information was available for 10 piggeries. Two hundred twenty-two piggeries had concrete run-out, 62 had pure pasture run-out, 21 had mixed run-out (concrete and pasture), five had another type of ground (e.g. chipped wood or gravel), and 12 did not answer the question. Herd size ranged from 2 to more than 1500 pigs, with an average of 303 animals per farm. The most represented breeds were Landrace and Large White pigs (L/LW; N = 275) followed by Duroc (N = 31) and Mangalitza (also called curly-hair hogs; N = 25). Concrete run-outs were fenced with concrete walls, metallic bars, wooden boards, wire mesh, or various combinations of those. In the case of pasture run-outs, fences consisted in electric wires (ranging from 20 cm to 60 cm height), wire mesh, wooden boards, metallic bars, or combinations. None of these pasture fences were fixed deep in the ground.

Eighty farmers (24.8%) reported contacts of any category. They mentioned at least one contact since they acquired the piggery, which could go back to the 1970’s. The frequency of contacts ranged from one in 39 years to 50 per year. From these 80 piggeries, the majority had a pure pasture run-out. Contacts were significantly more reported from piggeries with pasture and mixed run-out than with concrete run-out (P < 0.001 and P = 0.011, respectively). Overall, there was an average of 178 contacts of any type per year for a total of 322 outdoor farms, or 0.55 contact/farm/year (95% CI 0.50–0.61). Contacts were reported in all calendar seasons and in all 17 cantons with wild boar presence.

Cases of cross-breeding (or intra-specific hybridization) of wild boar with different pig breeds were reported in 17/322 piggeries (5.3%; seven of them had already been announced by game-wardens). Most of them were cross-bred with L/LW (N = 6) and Mangalitza sows (N = 6). Cross-breeding additionally occurred twice with a pure Duroc, once with a cross-bred Duroc x L/LW, once with a Pot-bellied pig, and once with a Minipig. In one of these piggeries holding Mangalitza, single and multiple births of cross-bred piglets following intrusions of wild boar or excursions of domestic sows have been observed (at least once a year for the past 3–4 years). In one instance, eight sows gave simultaneously birth to cross-bred piglets (P9, Table 1). Cross-breeding occurred more often with Mangalitza than L/LW (P = 0.0003)*.* Overall, there was an average of five mating events per year for a total of 85 farms with pure pasture or mixed run-out, which corresponded to an estimated rate of 0.06 mating/farm/year (95% CI 0.02–0.13). Cross-bred piglets were reported in 10 cantons, and in one case no information was obtained on piggery location.

**Table 1:** **Outdoor pigs from regions with wild boar presence sampled for serological and microbiological testing for*Brucella suis***

**Farm Nr**	**N pigs**	**Run-out**	**Farm type**	**Pig breed**	**Contact category**	**Particularities**	**N tested**	**Alive vs slaughtered**
**P1**	1000	Pasture	F^1^ + B^1^	L/LW^2^	3	Close contact with wild boar shedding *Brucella suis*	51/27 19	A^3^ / S^3^ A + S
**P2**	5	Concrete	F	L/LW	2		7	S
**P3**	9	Mixed	F	M^2^	1		4	S
**P4**	70	Pasture	F	L/LW	3		30	S
**P5**	45^4^	Pasture	F + B	M	4	Reproduction problems	5/6 16	A / S^5^ A + SO^3^
**P6**	12	Pasture	F + B	L/LW + hybrids^6^	1		3	S
**P7**	30	Pasture	F	M	None	Reproduction problems	14	A
**P8**	24	Pasture	F + B	M	4		2	A
**P9**	29	Pasture	F + B	M + hybrids	4		5	A
**P10**	14	Pasture	B	M + hybrids^7^	2		5/6	A / S
**P11**	23	Pasture + mixed	F + B	M	None^8^		3	A
**P12**	4	Concrete	B	M + Duroc	1	Boar borrowed from and sow lent to P5	4	A + S
**P13**	11	Pasture	F + B	M	None	Fattening pigs bought from *B. suis* infected piggery (P5)	6 5	A A + S

^1^ F: Fattening, B: Breeding.

^2^ L/LW: Large white or Landrace, M: Mangalitza.

^3^A: Alive, S: Slaughtered, SO: Stamped-out.

^4^Including 17 newborn piglets.

^5^Pigs slaughtered before the discovery of the outbreak.

^6^Hybrids were bought from another outdoor piggery for breeding purposes. In this other piggery, however, hybridization had been accidental (wild boar intrusion).

^7^A breeding domestic sow, which had mated with a wild boar (accidental intrusion), was bought pregnant of hybrid piglets from another outdoor piggery

^8^Sampling on request of the pig owner.

Sampled domestic pigs from Swiss outdoor piggeries (P) in 2008–2010 in a study on risk factors for contacts between wild boar and outdoor pigs. Categories of contacts: (1) presence of wild boar around the enclosure (> 2 m); (2) presence of wild boar just outside the fence (< 2 m); (3) wild boar intrusions without mating; and (4) mating (hybrid births).

### Surveillance of piggeries with camera traps

During one year of surveillance in two piggeries at risk (P1 and P4, Table 1), wild boar were detected only in piggery P1. There were four detections in nearly 16 months, always just outside the enclosure (< 2 m) on the pedestrian way between the forest and the fence, at night (between 6 p.m. and 2 a.m.), from December to March: (1) single juvenile; (2) wild sow with offspring; (3) single adult (sex not identifiable); (4) two animals (undetermined age and sex).

### Assessment of risk factors for contacts

Information on potential risk factors for contacts between wild boar and domestic pigs (Table 2) were obtained from 322 pig farmers. Selected factors included in the multivariable logistic regression models are listed in Additional file 1a-d. The final multivariable models, one for each outcome (Table 3, 4, 5, 6), showed that (1) pigs in enclosures separated from the piggery building (> 5 m) or/and located away from other houses (> 500 m) or/and close to a forest (< 500 m) were most at risk for indirect contacts with wild boar roaming around the piggery (2–500 m); (2) pigs in enclosures separated from the piggery building (> 5 m) or/and protected with an electric fence were most at risk for closer indirect contacts with wild boar (< 2 m from the fence), (3) pigs in enclosures located far from the piggery building (> 500 m) or/and protected by an electric fence or/and any fence **≤** 60 cm, were most at risk for an intrusion; and (4) piggeries holding Mangalitza or/and protected by an electric fence were most at risk for the occurrence of cross-breeding (i.e., for mating with wild boar). When comparing the most represented breeds with each other, cross-breeding was more frequent among Mangalitza than L/LW (odds ratio: 11.55, 95% CI_OR_ = 3.41–39.13).

**Table 2:** Explicative variables and hypotheses

**Variables**	**Hypotheses: situations increasing the risk of contacts between pigs and wild boars**
Run-out type: concrete vs mixed vs pure pasture	Pure pasture as run-out (is less protected than concrete or mixed run-out)
Fattenig vs mixed vs breeding farm	Breeding farm (presence of several sexually mature sows attractive for boars)
Corn culture	Corn culture next to the enclosure (food source)
Grass cutlure	Grass culture next to enclosure (food source)
Distance enclosure-farm < 5 m	The larger the distance between the pig enclosure and the farm buildings, the higher the risk (lower disturbance by human presence)
Distance enclosure-farm < 50 m	
Distance enclosure-farm < 100 m	
Distance enclosure-farm < 500 m	
Distance enclosure-houses < 5 m	The larger the distance between the pig enclosure and the houses, the higher the risk (lower disturbance by human presence)
Distance enclosure-houses < 50 m	
Distance enclosure-houses < 100 m	
Distance enclosure-houses < 500 m	
Distance enclosure-forest < 5 m	The shorter the distance between the pig enclosure and the forest, the higher the risk (proximity to wild boar habitat)
Distance enclosure-forest < 50 m	
Distance enclosure-forest < 100 m	
Distance enclosure-forest < 500 m	
Herd size: < 50 pigs vs > 50 pigs	Small herd size (less intimidating and thus more attracting to wild boar).
Breeding sow: absence vs presence	Presence of breeding sow in the enclosure (attractive to wild boar males)
Breeding hog: absence vs presence	Absence of breeding hog in the enclosure (presence is considered a protective factor)
Landrace/Large White vs other breeds vs Mangalitza	A specific pig breed may be more attractive to wild boars
Other animal species near the enclosure: absence vs presence	Absence of other animals near the enclosure (less disturbance)
Access to run-out whole year vs part year	Access of domestic pigs to run-out the whole year (higher exposure, in particular potential higher contact risk for sows during the wild boar rut)
Presence of farmer around the farm	Absence of farmer near the enclosure (lower disturbance by human presence)
Presence of walkers around the farm	Absence of walkers near the enclosure (lower disturbance by human presence)
Fence type: solid vs flexible	Flexible fence (solid walls provide better protection against wild boar intrusions)
Fence height: < 60 cm vs > 60 cm	Low fences (<60 cm; easily passed by wild boars)

Single variables and hypotheses regarding their influence on the occurrence of contacts between outdoor pigs and wild boar.

**Table 3:** Final multivariable model 1 for risk factors for contacts between outdoor pigs and wild boar in Switzerland

**Model 1 (N = 253, Pseudo-R**^**2**^ **= 0.78)**				
**Risk factors significantly associated with presence of wild boar around a farm (2–500 m)**				
		**p**	**OR**	**95% CI**_**OR**_
distance enclosure-farm	< 5 m	baseline		
	> 5 m	0.001	2.80	1.52–5.14
distance enclosure-houses	< 500 m	baseline		
	> 500 m	0.005	5.06	1.44–18.12
distance enclosure-forest	> 500 m	baseline		
	< 500 m	0.012	5.10	1.65–15.52

Multivariable logistic regression models of risk factors in a study performed in 2008–2010. Significant associations with wild boar contacts are expressed by odds ratios (OR) and respective 95% confidence intervals (95% CI). The number of records considered in each model is indicated in parentheses (N).

**Table 4:** Final multivariable model 2 for risk factors for contacts between outdoor pigs and wild boar in Switzerland

**Model 2 (N = 254, Pseudo-R**^**2**^ **= 0.98)**				
**Risk factors significantly associated with wild boar at the fence (0–2 m)**				
		**p**	**OR**	**95% CI**_**OR**_
distance enclosure-farm	< 5 m	baseline		
	> 5 m	0.026	2.68	1.12–6.38
fence type	solid fence	baseline		
	flexible fence	0.003	3.75	1.58–8.89

**Table 5:** Final multivariable model 3 for risk factors for contacts between outdoor pigs and wild boar in Switzerland

**Model 3 (N = 207, Pseudo-R**^**2**^ **= 0.76)**				
**Risk factors significantly associated with intrusion of wild boar**				
		**p**	**OR**	**95% CI**_**OR**_
distance enclosure-farm	< 500 m	baseline		
	> 500 m	0.036	8.28	1.15–59.73
fence type	solid fence	baseline		
	flexible fence	0.040	4.71	1.08–20.61
fence height	> 60 cm	baseline		
	< 60 cm	0.038	4.81	1.09–21.24

**Table 6:** Final multivariable model 4 for risk factors for contacts between outdoor pigs and wild boar in Switzerland

**Model 4 (N = 236, Pseudo-R**^**2**^ **= 0.97)**				
**Risk factors significantly associated with cross-breeding with wild boar**				
		**p**	**OR**	**95% CI**_**OR**_
breed	Large white/Landrace	baseline		
	Mangalitza	0.003	11.19	2.30–54.50
fence type	solid fence	baseline		
	flexible fence	0.003	10.49	2.18–50.48

### Testing for *B. suis* infection of exposed pigs

**Figure 1:**
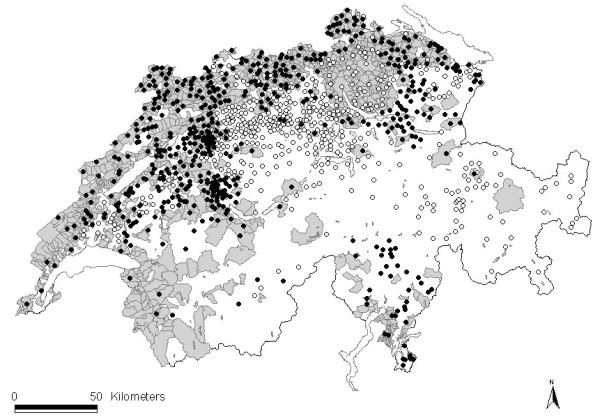
**Summary of information collected on the brucellosis outbreak in domestic pigs in Switzerland in 2009.** Piggeries where microbiological investigations could be carried out (P5, P12, P13) are numbered according to Table 1. Pink text boxes: piggery characteristics. Pig numbers are given for the time period of the outbreak. Dark blue text boxes: summary of unusual events noticed in the piggeries in 2008–2009. Light blue text boxes: results of bacteriological tests for *Brucella* sp. performed in the context of the outbreak. Yellow arrows show animal movements among piggeries.

Tested animals of 10/11 initially selected piggeries (P1-11, Table 1) were negative both by serology and qPCR, including the pigs from P1, which had been in close contact with an infected wild boar (for piggery history, see Methods). In parallel to these investigations, a recently acquired breeding boar (B2) from P5 died of a peritonitis thought to be due to a foreign body, and bacteriological culture performed post-mortem revealed a concurrent infection with *B. suis* Biovar 2 in the epididymidis [23]. All pigs older than six months subsequently tested seropositive to *B. suis* (both in the RBT and CFT) in this piggery were stamped out (Figure 1, P5). Six of 16 were positive by qPCR and five of them in culture [23]. Five qPCR-positive adult sows (of which four were also culture-positive) presented severe macroscopic lesions: metritis with miliary abscesses (N = 3), mastitis, and pyometra.

### Epidemiological investigations on the brucellosis outbreak in Mangalitza pigs

Animal movements from and to P5 and results of bacteriological investigations in the concerned piggeries are presented in Figure 1. Two potential sources of infection were considered for P5: (1) Spill-over from wild boar: births of cross-bred piglets in 2004 indicated successful mating between domestic sows and wild boar. A prevalence of 22.4% has been documented for *B. suis* in wild boar from this area, including adults shedding bacteria [1]. Animals had regularly been exchanged between P5 and another piggery (P12) since 2005, and health problems had been observed since 2008 in both P5 and P12. Therefore, P5 could have been infected by wild boar, and P12 by P5. P5 also infected P13 in selling fattening pigs. However, comparison of strains from selected wild boar, brown hares (*Lepus europaeus*) and affected pigs revealed distinct clusters, and wild boar were considered an unlikely source of infection [23]. (2) Introduction of infected pigs: While B2 was introduced after the decrease of reproduction and therefore considered an unlikely source of infection, P12 could have been contaminated by young piglets bought from other farms and have been the origin of the outbreak in P5.

## Discussion

A considerable geographical overlap between the wild boar population and outdoor piggeries in Switzerland was previously documented, pointing at a risk of contacts between wild boar and pigs [1]. Here, the occurrence of contacts was assessed, risk factors for these interactions were identified, and pigs from farms at risk were tested for *B. suis* as an example of pathogen transmissible from wild boar to pigs.

### Occurrence of contacts between wild boar and pigs

 To obtain a data set as complete as possible on the occurrence of contacts between wild boar and domestic pigs, we combined several methodical approaches. First, we carried out two parallel questionnaire surveys among different target groups, namely pig farmers (who are mostly present in proximity of pig enclosures but may not want to announce observed contacts by fear of sanctions by the local authorities) and game-wardens (who are usually called in case of wild boar intrusions and do not mind reporting such information). This proved to be efficient as obtained information was largely complementary. Because questionnaire surveys can be easily performed at a large scale, they give access to a large amount of data within a limited time period and increase the chance to record rare events. In a second step, we completed the questionnaire surveys by telephone interviews and visits of selected piggeries with the aim to validate information received in written form. Past and present occurrence of cross-bred animals was mostly recorded during such visits, revealing that cross-breeding was under-reported in mailed questionnaires filled by farmers. This is probably due to the fact that farmers are worried about the consequences it may have for their piggery if close contacts with wild boar are known to occur, such as the request of expensive fence transformations by the veterinary authorities or the loss of the official “piggery health status” attributed by the Swiss Pig Health Service. Personal contacts allow to gain the trust of the farmers and thus to obtain more truthful data. The final data set indicated that the observed yearly contact rate between wild boar and pigs per farm is low but that the number of observed contacts varies greatly among farms. Overall, contacts are not rare and sometimes even lead to cross-breeding.

Additionally, we surveyed two selected piggeries at risk with camera traps. Because this method is work-intensive and requests expensive material, it is not applicable on a large number of farms. Also, as contacts with wild boar on a single farm may not be frequent, the expected number of detections is disproportionally low compared to the invested effort. However, results of camera surveillance confirmed our assumption that many contacts are not observed by the farm personal since they occur at night, which is to be expected given that wild boar are mainly nocturnal [1]. Consequently, the derived contact rates are conservative since they underestimate reality.

Intrusions of wild boar and mating with domestic sows have been documented in France and Germany [22,24] but to our knowledge this study is the first to quantify contacts between pigs and wild boar including cross-breeding in piggeries. Established populations of hybrids are numerous in the United States [25], Sardinia [2], and also exist in southern Switzerland (canton of Ticino) [26]. However, these cross-bred animals are free-ranging and related to feral pigs and are relevant rather for conservation issues than for pig farming.

### Risk factors for contacts

This study is also the first one investigating risk factors for contacts between wild boar and domestic pigs. Because of the limited number of participants and the fact that some farmers had omitted to announce observed close contacts in the mail questionnaire, bias cannot be excluded. However, most identified factors corresponded to our expectations (pig enclosure location away from buildings and close to a forest, protection by low and flexible fences). The fact that risk factors for distant contacts were not necessarily identified for closer contact categories may have two explanations. Firstly, the backward selection procedure targeted the most significant factors for each category, some factors may have been eliminated even if playing a certain role once a strong factor was already part of the respective model. Secondly, data sets to a certain extent varied among contact categories, thus potentially influencing the weight of each considered factor. Nevertheless, a large distance to buildings of any kind and poor fence protection were repeatedly identified as risk factors in the final models. Overall, piggeries with concrete run-out (i.e., with solid fences such as metallic bars and concrete walls, and usually adjacent to buildings) are less at risk for any kind of contact with wild boar than piggeries with pasture run-out (which are mostly protected by electric fences and often located on pastures distant from the farm).

Interestingly, our analysis revealed that the pig breed is also important, Mangalitza being more at risk for mating with wild boar than other breeds. There is a higher proportion of Mangalitza pigs kept on pasture run-out than L/LW pigs, but these are generally closer to forested areas. Nevertheless, the final model showed that the enclosure location and the breed are independent risk factors and not confounders. In Switzerland, Mangalitza is the breed looking most similar and genetically most closely related to wild boar [27], and it could be more attracting to wild boar than other breeds.

### Infection status of exposed pigs

Pig owner compliance for visits and pig testing was moderate. This was partly due to the fact that positive results have to be announced to the veterinary authorities with severe consequences for the piggery. Only few piggeries and sometimes only a small proportion of the stock could be sampled but an outbreak of brucellosis was documented. The piggery first found to be infected (P5) gathered all identified risk factors and had experienced a hybrid birth, suggesting an infection through contacts with wild boar, as documented in other countries [22,24]. However, phylogenetic analysis of *B. suis* isolated from pigs and wild boar from the surrounding areas revealed distinct clusters and it was considered unlikely that wild boar were the direct origin of the outbreak, although possibly not all wildlife strains were included in the analysis [23]. To our knowledge, spill-over from wild boar to pigs has so far always been concluded from field evidence but not proven by strain analysis. Due to the regular pig exchanges between P5 and P12, it was not possible to determine which piggery had infected the other, and to date the source of infection remains unidentified.

### Risk of pathogen spill-over from wild boar to outdoor pigs

Wild boar can carry many pathogens and contacts between wild boar and pigs bear a considerable risk of spill-over [28,29]. Although the risk of transmission is expected to increase from category 1 to 4, it is largely dependent on the transmission route of the pathogens [4,30]. Thus, the risk of spill-over is highest for infectious agents transmitted by aerosols, to which all outdoor piggeries in areas with wild boar presence may be exposed (contact categories 1–4), whereas a spill-over is least likely for agents transmitted mainly via the venereal route.

Here, investigations focussed on *B. suis*, which is one of the wild boar pathogen of major concern for domestic pigs in Switzerland [1,20]. Although *B. suis* infection is widespread in Swiss wild boar and mating of wild boar with pig sows regularly occurs, a spill-over from wild boar to pigs could not be documented, suggesting that the risk of transmission of *B. suis* from wild boar to pigs is negligible. However, a spill-over cannot be completely ruled out because the number of sampled piggeries was low, mating had been documented in only few of them, and the origin of the brucellosis outbreak could not be definitely elucidated. Furthermore, while the risk of *B. suis* spill-over to pigs may have been very low up to now, the situation could change due to the significant increase of both wild boar abundance and prevalence of *B. suis* in wild boar [1].

### Prevention strategies

The outbreak was discovered in the frame of a research project. Brucellosis may be unapparent among living animals since infected sows can still give birth, though after few oestrous cycles of sexual rest [31], and infected boars can remain fertile [28]. Furthermore, metritis can easily be overlooked at slaughter, and infections are not always associated to pathological changes. Despite decreased reproduction success in two affected piggeries (P5, P12), veterinary investigations were not carried out and pigs were further exchanged. This indicates a need to increase the awareness of farmers for infectious diseases.

In Switzerland, targeted surveillance of porcine brucellosis is carried out only for breeding boars of conventional breeds. Mangalitza pigs are not represented in semen collection centres but boars are exchanged for mating, and they are not tested for brucellosis because Switzerland is considered free of this disease. It is therefore difficult to completely exclude *B. suis* infection in the pig population. Based on the present results, serological testing should be carried out before exchanging animals.

Our data indicate that outdoor piggeries with low electric fence (i.e., pasture run-out), located up to 500 m to a forest within the wild boar range and distanced from buildings, in particular those holding Mangalitza, are exposed to a serious risk of interactions with wild boar and need better protection. Significant seasonal differences regarding the spatial movements of wild boar were not observed [1] and contacts with domestic pigs were reported in all calendar season. Thus, this protection should be present the whole year round. Because wild boar seem to be generally more attracted by sows than by food [32,33], it seems appropriate to have the most reliable protection where mature sows are held. Most fences are not wild boar-proof, but wire-mesh fencing added to an electrified wire are most effective [34]. To avoid intrusions and escapes, the Swiss Pig Health Service recommends building two 1.50 m wire-mesh fences in parallel, fixed 30 cm deep in the ground to avoid the fence to be sapped, or reinforced by an additional electric fence around the pasture. However, the implementation of this type of fencing is challenging because it is perceived as not feasible by large piggeries regularly performing pasture rotations, both on a financial and practical point of view.

## Conclusions

This study shows that interactions between wild boar and outdoor pigs are not uncommon, pointing at the risk of pathogen spill-over even if a wild boar origin could not be documented in the case of the outbreak described here. It also provides data on risk factors that could help the risk-based implementation of protection measures for piggeries. Since a further augmentation of the wild boar population is expected [1] and modern animal welfare requirements are in favour of outdoor facilities for domestic livestock, interactions between wild boar and pigs will likely increase in the future. Our study underlines the importance of improving surveillance strategies for pathogens shared between wildlife and domestic animals and the need to increase disease awareness of farmers and veterinary practitioners.

## Methods

### Study area

The study was conducted in Switzerland (4284.57 km^2^) from March 2008 to March 2010. No reliable data are available on wild boar numbers but the hunting bag has shown an exponential increase in the past decade [1], reaching 6’878 hunted wild boar in 2010 [4]. Official statistics reported 1’588’998 domestic pigs in 2010 for 8’848 farmers [35], including 4’426 outdoor farms in 2008–2009 [1]. Wild boars are most widespread in the mountain range along the western and northern Swiss borders, while the majority of outdoor piggeries are located in central lowlands [1].

### Contacts between wild boar and domestic pigs

#### Definition of contact

Four categories of contacts were defined according to the risk of pathogen transmission: (1) presence of wild boar around the pig enclosure (2 m to 500 m; indicators: tracks, direct observations); (2) presence of wild boar just outside the fence (up to 2 m; indicators: tracks, direct observations); (3) wild boar intrusions into the pig enclosure without documented mating (indicators: direct observations); (4) mating following intrusions or escapes (indicators: births of cross-bred piglets).

### Questionnaire survey among game-wardens

In spring 2008, a questionnaire was sent to the wildlife services of all Swiss cantons, which are usually informed about wild boar intrusions into domestic pig enclosures. Game-wardens (or hunters, depending on the hunting system of the canton, but referred to as game-wardens here for simplification) with wild boar in their areas of surveillance were asked to report contacts observed within the past 15 years. Questions concerned the presence of outdoor pigs, and the occurrence, categories and frequency of contacts with wild boar. Responses were sent by 302 game-wardens from 15 cantons, while 11 cantons did not participate. Reasons for not participating were: absence of wild boar hordes in these regions, no wild boar hunt, or impossibility to provide data at the time of the study. Eighty-two questionnaires reporting presence of both wild boar and outdoor pigs were considered for this study, and 236 responses were excluded for the following reasons: no outdoor piggeries (N = 205), no wild boar in the region (N = 29) or incomplete questionnaires (N = 2).

### Questionnaire to owners of outdoors pigs

**Figure 2:**
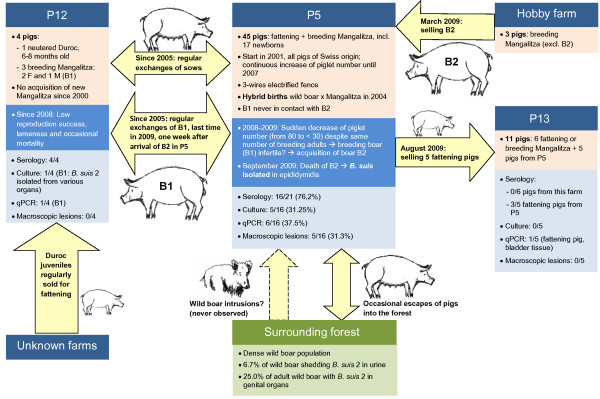
**Occurrence of wild boar and outdoor pigs, and location of selected pig farms.** Map of Switzerland showing communes with wild boar occurrence (grey areas) and outdoor piggeries (dots). Black dots: farms selected to study risk factors for contacts between wild boar and outdoor pigs in 2009–2010; white dots: not selected farms.

Subsequently, based on hypotheses regarding factors which could influence the occurrence of contacts between outdoor pigs and wild boar (Table 2), a more detailed questionnaire was designed for owners of outdoor pigs, asking about the location and characteristics of piggeries, and the occurrence and categories of contacts between pigs and wild boar (full questionnaire available from the corresponding author). The questionnaire was improved after interviews with two selected farmers and sent between January and July 2009 to all 1279 piggeries with concrete and pasture run-out located in Swiss communes with reported wild boar presence [1] (Figure 2), excepting the canton of Zurich, for which data were not available at the time of the survey. Thanks to telephone interviews and visits of selected farmers (see below), we additionally obtained information on known piggeries where contacts with wild boar had occurred but that were either not included in the official databases or had originally not been selected due to their location just outside regions at risk or which had initially not answered our questionnaire. These farms were then contacted for a telephone interview or personal visit. Participation was voluntary and data were treated anonymously.

Three hundred and seventy-one pig owners (29% response rate) participated to the survey; 322 responses were included in the study (including partially filled questionnaires) and 49 excluded (no pigs anymore, or no wild boar in the region). Missing information from 32 piggeries with reported contacts was gathered by telephone interviews.

To validate answers from completed questionnaires, selected piggeries were personally visited between April 2009 and March 2010. Following piggeries were contacted: all which announced contacts (N = 80), and a selection with pasture or mixed run-out but without observed contacts (N = 33); 48 owners gave their agreement (33 pure pasture run-out, 13 mixed pasture/concrete run-out, and two pure concrete run-out).

Finally, data obtained through both questionnaire surveys (farmers and game-wardens/hunters) were confronted to remove unnecessary data on contacts announced in both surveys, and data sets were merged. In the questionnaire, farmers could report observed contacts either as mean number of contacts per year or as total number of contact since they own the farm. An estimated contact rate per farm and year was obtained by first calculating the average number of recorded contacts per year for each farm (if not already indicated by the farmer), and subsequently summing these averages to obtain the average number (over all farms) of contacts per year.

### Surveillance of outdoor piggeries with camera traps

Two piggeries with L/LW on pure pasture run-out (piggeries P1 and P4, Table 1) which had participated in the questionnaire survey, were monitored with infrared camera traps (Reconyx 55, *www.reconyx.com*) to attempt to directly document contacts between wild boar and outdoor pigs at any time of the day and night over four seasons. In both piggeries, wild boar had been previously observed around and inside the enclosures situated between agricultural fields and a forest. The cameras, which detect movement up to 15.2 m away and record date and time of the pictures, were placed along the forest, in opposite direction to the enclosure. They shot five pictures per trigger with one second of delay between two triggers, and remained constantly active.

In P1, two to three cameras (depending on the location) were installed from December 2008 to March 2010, in three different locations due to pasture rotations, and for a duration of six, seven, and three months, respectively. This piggery was protected by a three-wires electrified fence (60 cm high). In P4, two cameras were installed from September to November 2009. The pasture was surrounded by a single-wire electrified fence additionally to a wire-mesh fence fixed above the ground. Animal number, date and age (defined according to Wu and others [1]) were recorded for each photographed wild boar.

### Statistical analyses for risk factors

For risk factor analyses, piggeries were considered “positive” for the closest reported contact category and all other more distant categories, e.g., piggeries with evidence of mating were also considered “positive” for the presence of wild boar around the enclosure and for wild boar intrusions, even if these contacts were not directly observed.

Analysis of risk factors for contacts between wild boar and domestic pigs was carried-out in a two-stage analysis with the NCSS 2007 software (Hintze J., 2006; NCSS, Kaysville, Utah, *www.ncss.com*). First, significant potential risk factors were separately identified for the four outcomes (different categories of contacts based on intensity) in a univariable approach, using Chi-square or Fisher’s exact test and logistic regression (Additional file 1a-d).

Subsequently, for each of the four outcomes (models) risk factors with statistical significance (P<0.05) at the univariable level were tested in a Spearman rank correlation matrix to identify and remove strongly correlated factors (rs ≥ 0.5; Additional file 1a-d) and therefore reduce the potential for multicollinearity in the final multivariable models [36]. For this procedure, contact categories were numerically coded according to the expected direction of effect (contact risk increase). Variables with a large proportion of missing values (e.g., pigs kept outside 24 h a day versus only part of the day, composition of wild boar group, wild boar sex and age category) or with imprecise records (e.g., seasonal occurrence and day time of different contact categories) were also excluded from the models.

The remaining variables were submitted to the second modeling step. Here final multivariable models (Table 3, 4, 5, 6) were obtained with a manual stepwise backward elimination procedure with a cut-off level at P < 0.05. A manual inclusion procedure was applied to test whether factors previously eliminated but considered important from a biological point of view would be significant in the final risk factor models. However, because none of these factors became significant, the final models arose from the stepwise backward elimination procedure only. Statistical significance of the risk factors in the models was determined by the Wald test.

### Survey for *B. suis* infection in selected outdoor piggeries

Following the questionnaire surveys and visits of piggeries, 11 farmers (piggeries P1-11, Table 1) gave their oral consent to let us sample their animals alive (breeding pigs) or at slaughter (fattening and finishing pigs). Sampling procedures were performed in accordance with the Swiss legislation. They were approved by the committee for animal experiments of the canton of Bern (authorization no. 102/09) and the concerned cantonal veterinary offices. Two piggeries that acquired pigs from a piggery infected with *B. suis* were additionally included in the study (P12-13, Table 1). Sampling related to the brucellosis outbreak was mandatory according to the Swiss legislation and performed under the supervision of the cantonal veterinary authorities. Altogether, 218 pigs were sampled: EDTA blood, serum and urine (whenever possible) were collected from all animals, and tissues (spleen, urinary bladder, uterus) were additionally taken from slaughtered pigs.

The majority of the tested pigs were from 10 piggeries with reported contacts with wild boar (Table 1). In P1, which reported regular wild boar presence around the pig enclosure and recurrent intrusions on the pasture, a wild boar shedding *B. suis* Biovar 2 in urine [1] had entered an enclosure of breeding sows and piglets for several hours in 2008. Blood samples (N = 46) and urine (N = 3) were subsequently collected from nine sows and 37 fattening pigs, which had been on the same pasture as the infected wild boar. All sows and 9/37 fattening pigs could be sampled alive twice at 90 days interval, as required in the Swiss federal ordinance on epizootics; 4/9 sows and 11/37 fattening pigs were sampled at slaughter. More pigs from P1 were sampled alive, at slaughter, or both (Table 1).

In living pigs, blood samples were taken on or close to the outdoor pasture by puncturing the jugular vein after manual contention of the animal by a pig farmer. Adults were immobilized with a snout rope placed behind the canine teeth, juveniles < 30 kg were placed in dorsal recumbency with their front limbs and head pulled cranially in order to better expose the jugular vein. Urine was collected with a plastic cup in case of spontaneous urination. Blood from slaughtered pigs was collected at the slaughterhouse with a plastic cup when carcasses were bled by the butcher. Urine was punctured from the urinary bladder immediately after extraction of the organs from the carcass. Tissue samples were also collected on site. After collection, urine samples were transferred into uncoated tubes, and blood samples into EDTA-coated and serum tubes. All samples were transported to the laboratory in a cool box. Serum tubes were centrifuged upon arrival and sera were immediately analyzed. All other samples were stored at −20°C until further analysis.

Sera were directly analysed with the rose bengal test (RBT) and with a complement fixation test (CFT), as recommended by the OIE [37]. Bacterial DNA was extracted with QIAamp® DNA Mini Kit (QIAGEN, Basel, Switzerland) from collected tissue samples and EDTA blood after thawing, and analysed by a qPCR targeting the *Brucella* spp. specific IS*711* insertion element [38].

## Competing interests

The authors declare that they have no competing interests.

## Authors’ contributions

NW carried out the questionnaire survey, farm visits, interviews and camera trapping, collected the blood and tissue samples, performed qPCR and part of the serological tests, contributed to field investigation of the brucellosis outbreak, analysed the data, and drafted the manuscript. CA and AT supervised the sampling of the pigs stamped out in the frame of the outbreak and the follow up of all laboratory work. PB performed the necropsy of the first pig affected by brucellosis and contributed to the isolation and analysis of this *Brucella* strain. EG contributed to sample collection and carried out most field investigations in the frame of the brucellosis outbreak. MD contributed to statistical analysis of risk factors. MPR designed the study, coordinated the project, contributed to sample collection, field investigations and data analysis, and drafted the manuscript. MPR and CA contributed to the acquisition of the funds. All authors have critically revised and approved the final version of the manuscript.

## Supplementary Material

Additional file 1 **1a-d - Univariable models for potential risk factors for the four categories of contacts between wild boar and outdoor pigs (.pdf).** Univariable association of potential risk factors in a study performed in Switzerland, 2009–2010. Significant associations with wild boar contacts are expressed by odds ratios (OR) and respective 95% confidence intervals (95% CI). Click here for file
