# Supplementary material for: Risk factors for contacts between wild boar and outdoor pigs in Switzerland and investigations on potential Brucella suis spill-over
Source: BMC Vet Res. 2012 Jul 20;8:116. doi: 10.1186/1746-6148-8-116 (PMC3464720; doi:10.1186/1746-6148-8-116)
Supplement: Additional file 1 — 1a-d - Univariable models for potential risk factors for the four categories of contacts between wild boar and outdoor pigs (.pdf). Univariable association of potential risk factors in a study performed in Switzerland, 2009–2010. Significant associations with wild boar contacts are expressed by odds ratios (OR) and respective 95% confidence intervals (95% CI). [file 1746-6148-8-116-S1.pdf]

Additional file 1a

Risk factors		p	OR	95% CI $_{OR}$
Significantly associated with (1) presence of wild boar around a farm (2-500 m):				
Presence of grass field	no	baseline		
	yes	0.021	2.41	1.13-5.15
Distance enclosure-farm	< 5m	baseline		
	> 5m	< .0001	3.26	1.86-5.72
Distance enclosure-farm	< 100m	baseline		
	> 100m	< .0001	5.40	2.32-12.56
Distance enclosure-houses	< 500m	baseline		
	> 500m	0.005	5.26	1.65-16.71
Distance enclosure-forest	> 50m	baseline		
	< 50m	0.006	2.48	1.29-4.80
Distance enclosure-forest	> 500m	baseline		
	< 500m	0.002	4.70	1.62-13.64
Herd size	< 50 pigs	baseline		
	> 50 pigs	0.009	2.06	1.19-3.55
Fence type	solid fence	baseline		
	flexible fence	< .0001	5.00	2.53-9.87
Fence height	> 60cm	baseline		
	< 60cm	0.0002	4.17	1.89-9.22

Not selected risk factors:
fattening farm vs other types of management, corn culture, distance enclosure-farm < 500m, distance enclosure-houses < 5m, distance enclosure-houses < 50m, distance enclosure-houses < 100m, distance enclosure-forest < 5m, distance enclosure-forest < 500m, presence of breeding sow, presence of breeding hog, presence of other animals near the enclosure, presence of farmer around the farm, presence of walkers around the farm, Large white/Landrace vs other breeds, Large white/Landrace vs Mangalitza

Eliminated factors due to correlation:
concrete vs other run-out correlated with distance enclosure-farm < 5 m, herd size, fence type
distance enclosure-farm < 50 m correlated with distance enclosure-farm < 100 m
distance enclosure-forest < 100 m correlated with distance enclosure-forest < 50 m

Additional file 1b

**Risk factors**		**p**	**OR**	**95% CI $_{OR}$**
Significantly associated with (2) wild boar at the fence (0-2 m):				
Distance enclosure-farm	< 5 m	baseline		
	> 5 m	0.001	3.80	1.68-8.60
Distance enclosure-farm	< 100 m	baseline		
	> 100 m	< .0001	8.25	3.35-20.34
Distance enclosure-farm:	< 500 m	baseline		
	> 500 m	< .0001	23.04	5.59-94.96
Herd size	< 50 pigs	baseline		
	> 50 pigs	0.037	2.19	1.04-5.64
Pig breed	Large white/Landrace	baseline		
	other breeds	0.042	1.40	0.54-3.67
	Mangalitza	0.042	3.27	1.25-8.57
Fence type	solid fence	baseline		
	flexible fence	< .0001	5.90	2.65-13.11
Fence height	> 60 cm	baseline		
	< 60 cm	0.001	5.29	2.14-13.11

Not selected risk factors:
fattening farm vs other management types, corn culture, grass culture, distance enclosure-houses < 5 m, distance enclosure-houses < 50 m, distance enclosure-houses < 100 m, distance enclosure-houses < 500 m, distance enclosure-forest < 5 m, distance enclosure-forest < 50 m, distance enclosure-forest < 100 m, distance enclosure-forest < 500m, presence of breeding sow, presence of breeding hog, presence of other animals near the enclosure, presence of farmer around the farm, presence of walkers around the farm

Eliminated factors due to correlation:
concrete vs other run-out correlated with distance enclosure-farm < 5 m, herd size, fence type
distance enclosure-farm < 50m correlated with distance enclosure-farm < 100 m

Additional file 1c

**Risk factors**		**p**	**OR**	**95% CI $_{OR}$**
Significantly associated with (3) intrusion of wild boar:				
Distance enclosure-farm	< 5m	baseline		
	> 5m	0.001	6.95	1.95-24.81
Distance enclosure-farm	< 100m	baseline		
	> 100m	< .0001	15.68	5.37-45.82
Distance enclosure-farm	< 500m	baseline		
	> 500m	0.0001	20.75	5.28-81.51
Fence type	solide fence	baseline		
	flexible fence	< .0001	14.00	5.24-42.74
Fence height	> 60cm	baseline		
	< 60cm	0.001	11.58	3.68-36.44
Not selected risk factors:<br>fattening farm vs other types of management, corn culture, grass culture, distance enclosure-houses < 5 m, distance enclosure-houses < 50 m, distance enclosure-houses < 100 m, distance enclosure-houses < 500 m, distance enclosure-forest < 5 m, distance enclosure-forest < 50 m. distance enclosure-forest < 100 m, distance enclosure-forest < 500 m, herd size, presence of breeding sow, presence of breeding hog, Large white/Landrace vs other breeds, Large white/Landrace vs Mangalitza, presence of other animals near the enclosure, presence of farmer around the farm, presence of walkers around the farm<br><br>Eliminated factors due to correlation:<br>concrete vs other run-out correlated with distance enclosure-farm < 5 m, herd size, fence type<br><br>distance enclosure-farm < 50 m correlated with distance enclosure-farm < 100 m				

Additional file 1d

**Risk factors**		**p**	**OR**	**95% CI $_{OR}$**
Significantly associated with (4) cross-breeding with wild boar:				
Distance enclosure-farm	< 100m	baseline		
	> 100m	0.001	11.43	3.06-42.67
Distance enclosure-forest	> 5m	baseline		
	< 5m	0.019	15.12	2.41-95.02
Distance enclosure-forest	> 50m	baseline		
	< 50m	0.013	6.34	1.02-39.20
Breed	Large white/Landrace	baseline		
	other breeds	< .0001	4.94	1.44-16.96
	Mangalitza	< .0001	11.55	3.41-39.13
Fence type	solid fence	baseline		
	flexible fence	0.0003	11.37	3.17-40.81
Fence height	> 60cm	baseline		
	< 60cm	0.005	0.01	2.08-28.40

Not selected risk factors:
fattening farm vs other, corn culture, grass culture, distance enclosure-farm < 5 m, distance enclosure-farm < 500 m, distance enclosure-houses < 5 m, distance enclosure-houses < 50 m, distance enclosure-houses < 100 m, distance enclosure-houses < 500 m, distance enclosure-forest < 500 m, herd size, breeding sow, breeding hog, presence of other animals near the enclosure, presence of farmer around the farm, presence of walkers around the farm

Eliminated factors due to correlation:
concrete vs other run-out correlated with distance enclosure-farm < 5 m, herd size, fence type
distance enclosure-farm < 50 m correlated with distance enclosure-farm < 100 m
distance enclosure-forest < 100 m correlated with distance enclosure-forest < 50 m
